# Prognosis of chronic kidney disease with normal-range proteinuria: The CKD-ROUTE study

**DOI:** 10.1371/journal.pone.0190493

**Published:** 2018-01-17

**Authors:** Soichiro Iimori, Shotaro Naito, Yumi Noda, Hidehiko Sato, Naohiro Nomura, Eisei Sohara, Tomokazu Okado, Sei Sasaki, Shinichi Uchida, Tatemitsu Rai

**Affiliations:** 1 Department of Nephrology, Tokyo Medical and Dental University, Bunkyo-ku, Tokyo, Japan; 2 Department of Nephrology, Nitobe Memorial Nakano General Hospital, Nakano-ku, Tokyo, Japan; The University of Tokyo, JAPAN

## Abstract

**Background:**

Although lower estimated glomerular filtration rate (eGFR) and higher proteinuria are high risks for mortality and kidney outcomes, the prognosis of chronic kidney disease (CKD) in patients with normal-range proteinuria remains unclear.

**Methods:**

In this prospective cohort study, 1138 newly visiting stage G2–G5 CKD patients were stratified into normal-range and abnormal-range proteinuria groups. Study endpoints were CKD progression (>50% eGFR loss or initiation of dialysis), cardiovascular events, and all-cause death.

**Results:**

In total, 927 patients who were followed for >6 months were included in the analysis. The mean age was 67 years, and 70.2% were male. During a median follow-up of 35 months, CKD progression, cardiovascular events, and mortality were observed in 223, 110, and 55 patients, respectively. Patients with normal-range proteinuria had a significantly lower risk for CKD progression (hazard ratio, 0.20; 95% confidence interval, 0.10–0.38) than those with abnormal-proteinuria by multivariate Cox proportional hazard analysis. We also analyzed patients with normal-range proteinuria (n = 351). Nephrosclerosis was the most frequent cause of CKD among all patients with normal-range proteinuria (59.7%). During a median follow-up of 36 months, CKD progression, cardiovascular events, and mortality were observed in 10, 28, and 18 patients, respectively. The Kaplan–Meyer analysis demonstrated that the risks of CKD progression and cardiovascular events were not significantly different among CKD stages, whereas the risk of death was significantly higher in patients with advanced-stage CKD. Multivariate Cox proportional hazard analysis showed that the risk of three endpoints did not significantly differ among CKD stages.

**Conclusion:**

Newly visiting CKD patients with normal-range proteinuria, who tend to be overlooked during health checkups did not exhibit a decrease in kidney function even in advanced CKD stages under specialized nephrology care.

## Introduction

Higher levels of albuminuria are widely demonstrated to precede and predict a faster rate of renal functional decline and to be associated with increased risk of end-stage kidney disease (ESKD), both in general population and in those with various pathophysiologic conditions such as diabetes, hypertension, and primary glomerular diseases [1]. Large meta-analyses also showed that lower estimated glomerular filtration rate (eGFR) and higher albuminuria were associated with several adverse outcomes [2, 3]. Findings from experimental and clinical studies suggest an important role for proteinuria in the pathogenesis of disease progression in chronic kidney disease (CKD) [4].

In Japan, the Industrial Safety and Health Law requires employees to undergo medical examination at least once a year. Furthermore, specific health checkups are available to medical insurance subscribers aged 40–74 years. These health checkups involve a dipstick urinary test; however, measurements for urinary protein, urinary albumin, or serum creatinine levels are not included. In a study including data from 574,024 participants (240,594 males and 333,430 females) over the age of 20 years that were from the Japanese general adult population, prevalence of patients with a eGFR of less than 60 ml/min/1.73 m^2^ and a negative proteinuria by dipstick urinary test was about 10%; such patients will be overlooked by only using dipstick test [5]. This finding raises a clinical question. Although lower eGFR and higher proteinuria indicate a high risk for mortality and worse kidney outcomes, the prognosis of CKD patients with normal-range proteinuria who may be overlooked during a health checkup, remains uncertain. Therefore, we aimed in this study to characterize the renal prognosis of CKD in patients with normal-range proteinuria under specialized nephrology care.

## Materials and methods

### Study design and cohort participants

The Chronic Kidney Disease Research of Outcomes in Treatment and Epidemiology (CKD-ROUTE) was a prospective, observational cohort study of a representative Japanese population with stage G2–G5 CKD according to the Kidney Disease Improving Global Outcomes (KDIGO) classification who were not undergoing dialysis [6]. Details of the study design were previously reported [7, 8]. Over 1,000 participants were enrolled at the Tokyo Medical and Dental University Hospital and its 15 affiliated hospitals in the Tokyo metropolitan area of Japan. At recruitment, written informed consent was obtained from all patients, and their eligibility was determined. This study was approved by the ethical committees of Tokyo Medical and Dental University, School of Medicine (No. 883) and all institutions participating in the study and was conducted in accordance with the ethical principles of the Declaration of Helsinki. The protocol was registered in UMIN Clinical Trials Registry (UMIN000004461).

Participants were eligible for inclusion if they (1) were newly visiting or were referred to the participating nephrology centers for the first time between October 2010 and December 2011, (2) were over 20 years of age, and (3) had stage G2–G5 CKD. The following patients were excluded from this study: (1) patients with malignancy that was diagnosed or treated within the previous two years; (2) transplant recipients; (3) patients with active gastrointestinal bleeding at enrollment; and (4) patients who did not provide written informed consent.

### Measurements

At the time of enrollment, medical history and current medications of all patients were recorded. Blood and urine samples were collected to measure clinical variables [7]. eGFR was calculated using the modified three-variable Modification of Diet in Renal Disease equation developed by the Japanese Society of Nephrology, to adjust for Japanese physical characteristics [9]: eGFR = 194 × serum creatinine ^−1.094^ × age ^−0.287^ (if female, × 0.739). Proteinuria was measured by both the dipstick test and the urinary protein/creatinine ratio (UPCR). Participants visited the hospital every 6 months for assessment of their clinical status. All participants were treated according to standard treatment protocols recommended by the Japanese CKD guidelines [10].

### Definitions of normal-range or abnormal-range proteinuria and cardiovascular disease

Normal-range proteinuria was defined as negative or trace protein by dipstick urinary test at enrollment. Abnormal-range proteinuria was defined as positive proteinuria by the dipstick urinary test. A history of cardiovascular disease (CVD) and cardiovascular events were defined as a history of one or more of the following diseases: ischemic heart disease (angina pectoris or myocardial infarction), congestive heart failure, peripheral arterial disease (necrosis, amputation, or revascularization surgery of the extremities), or stroke (cerebral infarction, transient ischemic attack, cerebral hemorrhage, or subarachnoid hemorrhage).

### Study endpoints

Primary endpoints were CKD progression defined as >50% eGFR loss or initiation of dialysis, cardiovascular events, and all-cause death. Cardiovascular events included ischemic heart disease, congestive heart failure, peripheral arterial disease, or stroke.

### Statistical analysis

Baseline characteristics were presented as means ± standard deviation or median and interquartile ranges (IQR) for continuous variables, whereas categorical data were presented as numbers and percentages. Comparison of baseline characteristics between the patients with proteinuria within normal-range and those with abnormal-range was evaluated using unpaired Student’s *t* test or Mann-Whitney U test as appropriate. Categorical data between the two groups were compared using the chi-squared test. Analysis of variance and chi-squared test were used to compare continuous and categorical variables across CKD stages in patients with normal-range proteinuria, respectively. Changes in eGFR during the study period were analyzed using repeated measures analysis of variance, followed by Bonferroni post hoc test. Differences in survival were compared using the Kaplan–Meier methods and the log-rank test. Cox proportional hazards model was used to estimate hazard ratios (HRs) of endpoints. Covariates that were significantly different between the groups at baseline were used to adjust HRs. Statistical analyses were performed using SPSS version 20.0 (Chicago, IL, USA). *P* values of <0.05 were considered statistically significant.

## Results

### Stratification of the CKD-ROUTE study participants stratified by eGFR and proteinuria categories

Classification of 1,138 newly visiting patients according to the severity of CKD is shown in Table 1 [7]. The percentage of patients with an eGFR of less than 60 ml/min/1.73 m^2^ and negative or trace proteinuria by dipstick urine test was 32.5%. For comparison, the classification of the study cohort according to the Japanese CKD guidelines is shown in S1 Table [10]; the percentage of patients with eGFR less than 60 ml/min/1.73 m^2^ and a UPCR of less than 0.15 g/gCr was 23.8%.

**Table 1:** Classification of chronic kidney disease by dipstick urine test for proteinuria.

			Dipstick test for proteinuria categories			Total
			(–) or trace	(+)	≥ (2+)	
			n (%)	n (%)	n (%)	
GFR categories (mL/min/1.73 m^2^)	G2	60–89	48 (4.3)	15 (1.3)	32 (2.9)	95 (8.5)
	G3a	45–59	105 (9.4)	30 (2.7)	52 (4.6)	187 (16.7)
	G3b	30–44	142 (12.7)	40 (3.6)	95 (8.5)	277 (24.7)
	G4	15–29	101 (9.0)	49 (4.4)	209 (18.6)	359 (32.0)
	G5	<15	16 (1.4)	29 (2.6)	159 (14.2)	204 (18.2)
Total			412 (36.7)	163 (14.5)	547 (48.8)	1122 (100)

Data are presented as numbers and percentages.

Abbreviations: GFR, glomerular filtration rate

### Eligibility criteria

**Fig 1:**
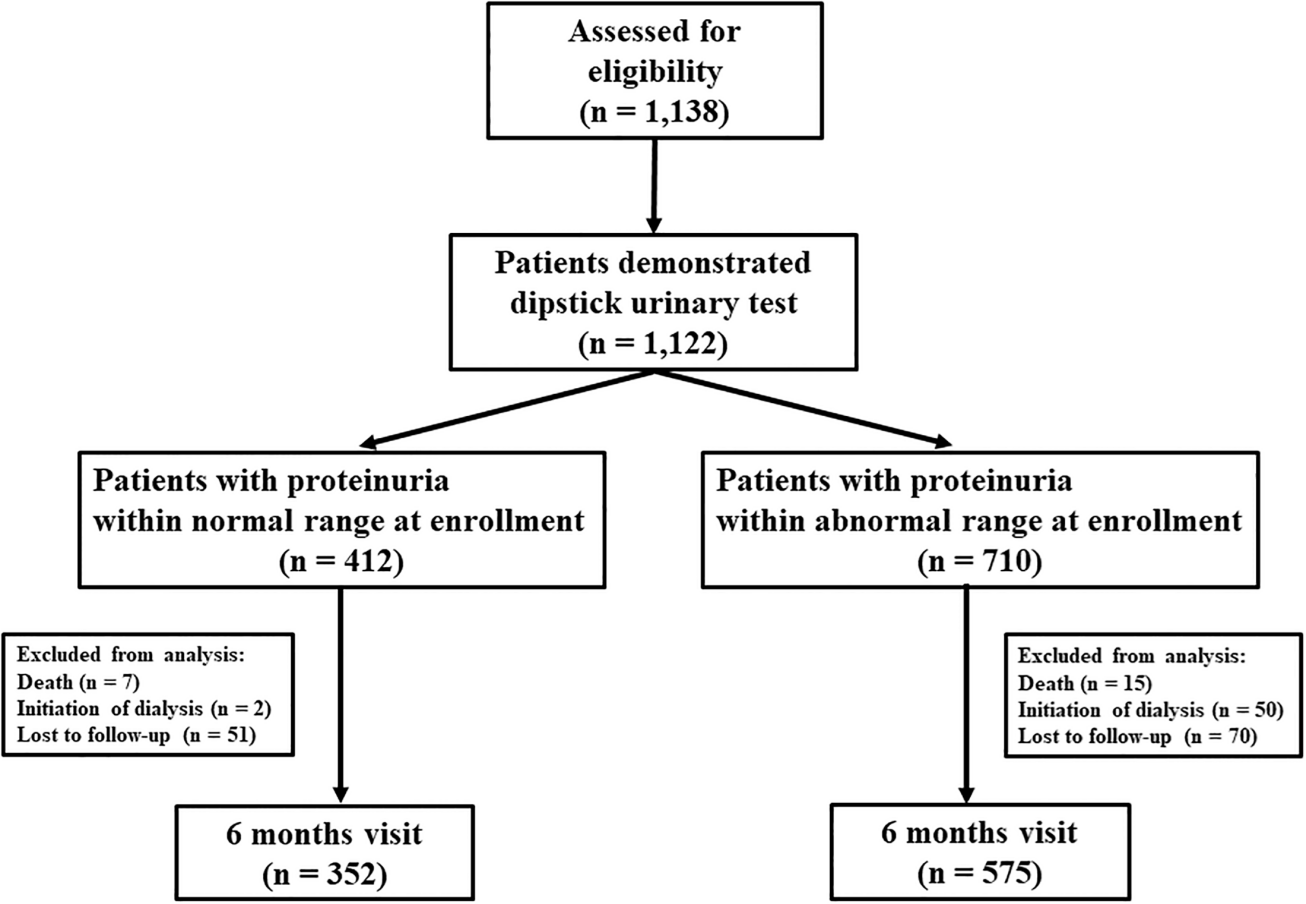
Flow chart of the study design.

Fig 1 shows the flow chart of the study design. In total 1,138 patients were assessed for eligibility, and 1,122 patients were evaluated by the dipstick urinary test. Patients were divided into normal-range and abnormal-range proteinuria groups at enrollment. Finally, 927 patients who were followed for >6 months were included in the final analysis (432 and 575 patients with normal-range and abnormal-range proteinuria, respectively).

### Prognosis of CKD patients with normal-range proteinuria compared with abnormal-range proteinuria

Table 2 shows the baseline characteristics of all patients at enrollment (n = 927). Mean age was 67 years, and 70.2% of the patients were male. Compared to the patients with abnormal-range proteinuria, those with normal-range proteinuria were older; had a lower body mass index; and had a lower prevalence of hypertension, diabetes, and CVD history (*P* < 0.05). Furthermore, patients with normal-range proteinuria had higher hemoglobin, higher eGFR, and a lower prevalence of RAAS inhibitor use (*P* < 0.01).

**Table 2:** Baseline characteristics of all the patients at enrollment (n = 927).

	all patients	normal-range proteinuria						abnormal-range proteinuria	
		total	G2	G3a	G3b	G4–G5	*P* value	total	*P* value (vs normal-range proteinuria)
	n = 927	n = 352	n = 36	n = 89	n = 129	n = 98		n = 575	
Age, y	67 ± 14	69 ± 13	60 ± 15	65 ± 13	71 ± 10	75 ± 11	<0.001	66 ± 14	<0.001
Male gender, n (%)	651 (70.2)	244 (69.3)	20 (55.6)	71 (79.8)	92 (71.3)	61 (62.2)	0.016	407 (70.8)	0.632
Systolic blood pressure, mmHg	139 ± 22	133 ± 20	140 ± 19	134 ± 20	130 ± 19	133 ± 21	0.059	143 ± 22	<0.001
Body mass index, kg/m^2^	23.8 ± 3.9	23.4 ± 3.6	22.4 ± 4.0	23.7 ± 3.3	23.3 ± 3.4	23.5 ± 3.9	0.4	24.0 ± 4.1	0.026
Etiology of CKD									<0.001
Diabetic nephropathy, n (%)	224 (24.2)	27 (7.7)	3 (8.3)	1 (1.1)	10 (7.8)	13 (13.3)	<0.001	197 (34.3)	
Nephrosclerosis, n (%)	376 (40.6)	210 (59.7)	12 (33.3)	49 (55.1)	80 (62.0)	69 (70.4)		166 (28.9)	
Glomerulonephritis, n (%)	174 (18.8)	24 (6.8)	9 (25.0)	8 (9.0)	5 (3.9)	2 (2.0)		150 (26.1)	
Other, n (%)	153 (16.5)	91 (25.9)	12 (33.3)	31 (34.8)	34 (26.4)	14 (14.3)		62 (10.8)	
Hypertension, n (%)	831 (89.6)	288 (81.8)	22 (61.1)	63 (70.8)	111 (86.0)	92 (93.9)	<0.001	543 (94.4)	<0.001
Diabetes, n (%)	341 (36.8)	94 (26.7)	5 (13.9)	13 (14.6)	35 (27.1)	41 (41.8)	<0.001	247 (43.0)	<0.001
History of CVD, n (%)	244 (26.3)	74 (21.0)	3 (8.3)	8 (9.0)	32 (24.8)	31 (31.6)	<0.001	170 (29.6)	0.004
Hemoglobin, g/dL	12.1 ± 2.2	12.7 ± 2.0	13.8 ± 1.6	13.6 ± 1.7	12.9 ± 1.9	11.2 ± 1.7	<0.001	11.8 ± 2.2	<0.001
Serum albumin, g/dL	3.9 ± 0.6	4.2 ± 0.4	4.3 ± 0.5	4.3 ± 0.4	4.2 ± 0.4	4.0 ± 0.4	<0.001	3.7 ± 0.6	<0.001
eGFR (ml/min/1.73m^2^)	33.8 ± 17.8	40.3 ± 15.6	69.9 ± 7.8	51.5 ± 4.1	38.2 ± 4.6	22.0 ± 5.1	<0.001	29.8 ± 18.0	<0.001
UPCR, g/gCr	0.64 [0.64–2.55]	0.08 [0.03–0.18]	0.07 [0.04–0.15]	0.06 [0.03–0.14]	0.08 [0.03–0.18]	0.12 [0.04–0.27]	0.022	1.72 [0.74–4.20]	<0.001
Urinary occult blood, n (%)	292 (31.5)	57 (16.2)	11 (30.6)	16 (18.0)	20 (15.5)	10 (10.2)	0.04	235 (40.9)	<0.001
Use of RAAS inhibitor, n (%)	601 (64.8)	206 (58.5)	7 (19.4)	38 (42.7)	86 (66.7)	75 (76.5)	<0.001	395 (68.7)	0.002
Use of calcium channel blocker, n (%)	440 (47.5)	119 (33.8)	1 (2.8)	29 (32.6)	49 (38.0)	40 (40.8)	<0.001	321 (55.8)	<0.001
Use of diuretics, n (%)	295 (31.8)	87 (24.7)	0 (0)	12 (13.5)	31 (24.0)	44 (44.9)	<0.001	208 (36.2)	<0.001

Continuous variables are presented as mean ± standard deviation and median with interquartile ranges. Categorical data are presented as numbers and percentages.

Abbreviations: CKD, chronic kidney disease; CVD, cardiovascular disease; eGFR, estimated glomerular filtration rate; UPCR, urinary protein/creatinine ratio; g/gCr, gram per gram creatinine; RAAS, renin angiotensin aldosterone system.

During a median follow up of 35 months, 223 patients had CKD progression, 110 patients had cardiovascular events, and 55 patients died. Table 3 shows the Cox proportional hazard analysis for disease endpoints. Univariate analysis to determine the risk of disease endpoints showed that patients with normal-range proteinuria were at low risk for CKD progression [hazard ratio (HR), 0.07; 95% confidence interval (95% CI), 0.03–0.12] and cardiovascular events (HR, 0.50; 95% CI, 0.32–0.80). However, all-cause death was not significantly different between the two groups (HR, 0.72; 95% CI, 0.41–1.26). Multivariate analysis showed that the risk of cardiovascular events was canceled in model 2. Patients with normal-range proteinuria had a significantly low risk for CKD progression (HR, 0.20; 95% CI, 0.10–0.38) by multivariate analysis in model 2.

**Table 3:** Cox proportional hazard model for risk of disease endpoints in all study participants (n = 927).

	Number of Incidence, n (%)		Unadjusted	Model 1	Model 2
	normal-range proteinuria	abnormal-range proteinuria			
	(n = 352)	(n = 575)	HR (95%CI)	HR (95%CI)	HR (95%CI)
CKD progression	10 (2.8)	213 (37.0)	0.07 (0.03–0.12)	0.08 (0.04–0.14)	0.20 (0.10–0.38)
Cardiovascular events	28 (8.0)	82 (14.3)	0.50 (0.32–0.80)	0.52 (0.33–0.80)	0.73 (0.45–1.19)
All-cause death	18 (5.1)	37 (6.4)	0.72 (0.41–1.26)	0.59 (0.33–1.05)	1.07 (0.56–2.03)

Model 1: Adjusted by age, gender, hypertension, diabetes, and history of CVD. Model 2: Adjusted by Model 1, hemoglobin, serum albumin, and the use of RAAS inhibitor.

Abbreviations: HR, hazard ratio; CI, confidence interval.

### Prognosis of CKD patients with normal-range proteinuria according to the CKD stage

Next, we analyzed CKD patients with normal-range proteinuria. Table 2 shows baseline characteristics of 352 patients with normal-range proteinuria stratified according to the CKD stage. Mean age was 69 years, and those with advanced-stage CKD were older (*P* < 0.001). Majority of the patients were male (69%), which was most frequent among those with stage G3a CKD. Nephrosclerosis was the most frequent cause of CKD among all patients (59.7%). CVD was prevalent in 21% of the entire cohort, and patients with advanced CKD were more likely to have CVD (*P* < 0.001). Mean hemoglobin and serum albumin levels were 12.7 and 4.2 g/dL, respectively, which decreased with CKD progression (*P* < 0.001). UPCR was slightly higher in patients with advanced CKD (*P* < 0.022). The percentage of patients with occult urinary blood was highest in those with stage G2 CKD (*P* = 0.04).

**Fig 2:**
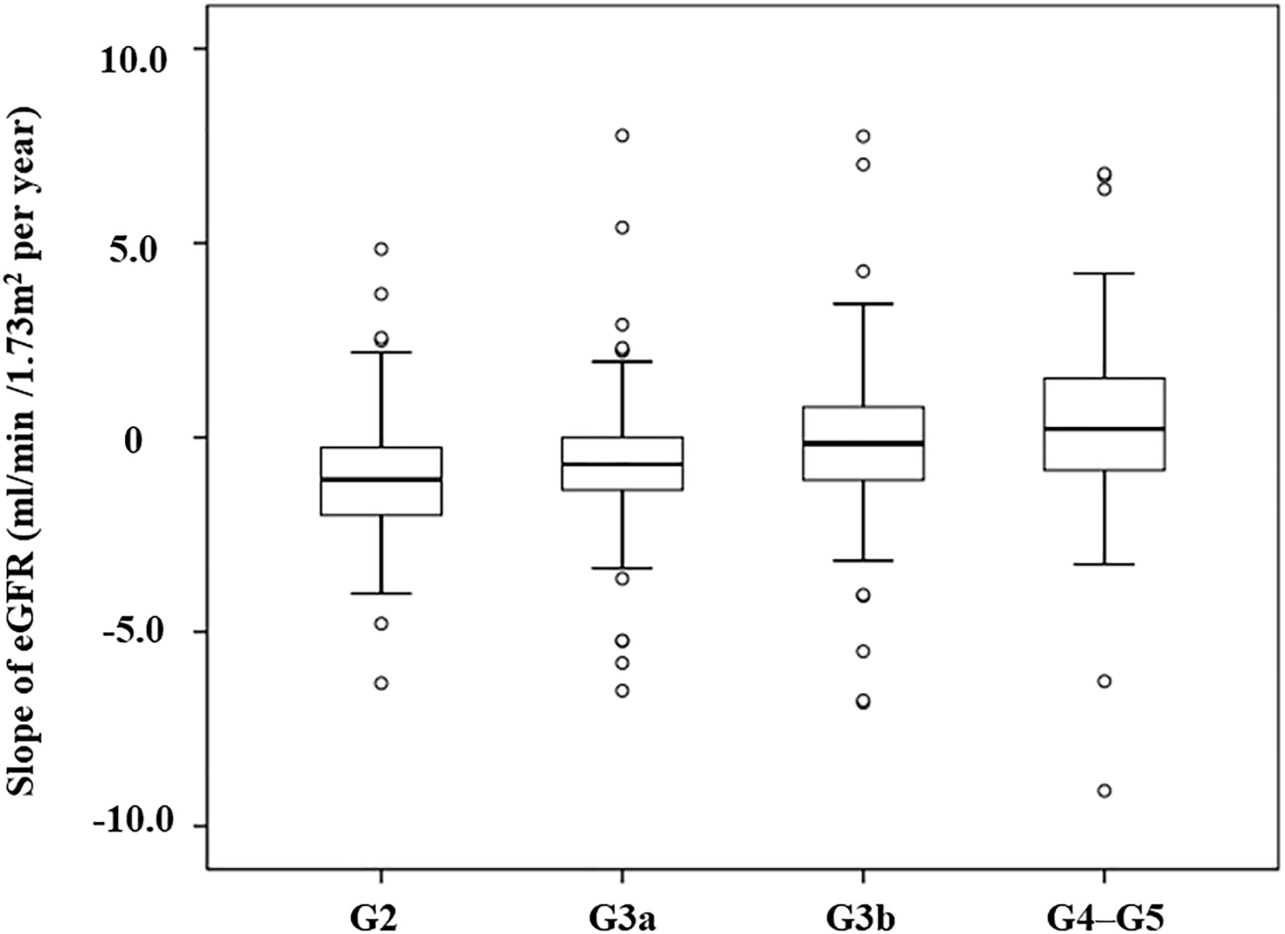
Slope of estimated glomerular filtration rate (eGFR) in patients with normal-range proteinuria from enrollment to the last visit (n = 352). Box and whisker plot (box represents the interquartile range; whiskers extend to the most extreme data point, which is no more than 1.5 times the interquartile range from the box; circles beyond the whiskers are extreme values; and the line within the box represents the median) represent changes in eGFR from baseline to the last visit.

During a median follow-up of 36 months, we calculated the slope of eGFR from enrollment to the last visit. In all patients (n = 352), the median eGFR decline was −0.69 (IQR, −2.53 to 1.65) ml/min/1.73m^2^ per year. Regarding CKD stages, G2, G3a, G3b, and G4–G5, the median eGFR declines were −2.17 (IQR, −3.99 to −0.51) ml/min/1.73m^2^ per year, −1.38 (IQR, −2.71 to 0) ml/min/1.73m^2^ per year, -0.31 (IQR, −2.19 to 1.57) ml/min/1.73m^2^ per year, and 0.44 (IQR, −1.69 to 3.05) ml/min/1.73m^2^ per year, respectively (Fig 2).

**Fig 3:**
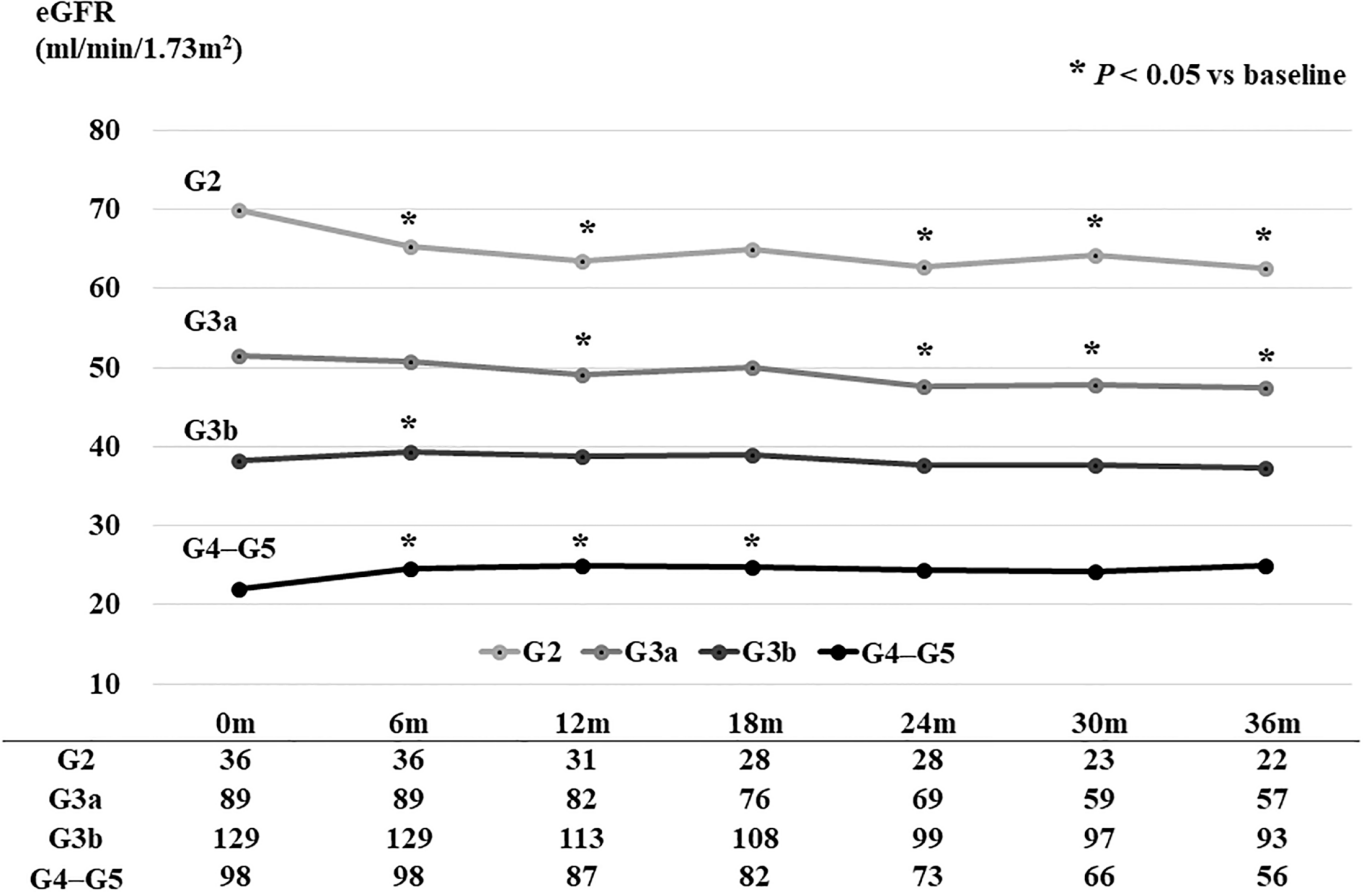
Change in estimated glomerular filtration rate (eGFR) in patients with normal-range proteinuria during 36 months of follow-up (n = 352). eGFR levels of stage G2 and G3a chronic kidney disease (CKD) patients significantly decreased, whereas those of patients with a stage more advanced than G3b did not decrease during 36 months of follow-up.

Changes in eGFR during the study period according to the CKD stage are shown in Fig 3. Briefly, eGFR levels decreased significantly in patients with stage G2 or G3a CKD (*P* < 0.05), whereas eGFR levels did not change in those with the advanced stages G3b or more over the course of a 36-month follow-up.

**Fig 4:**
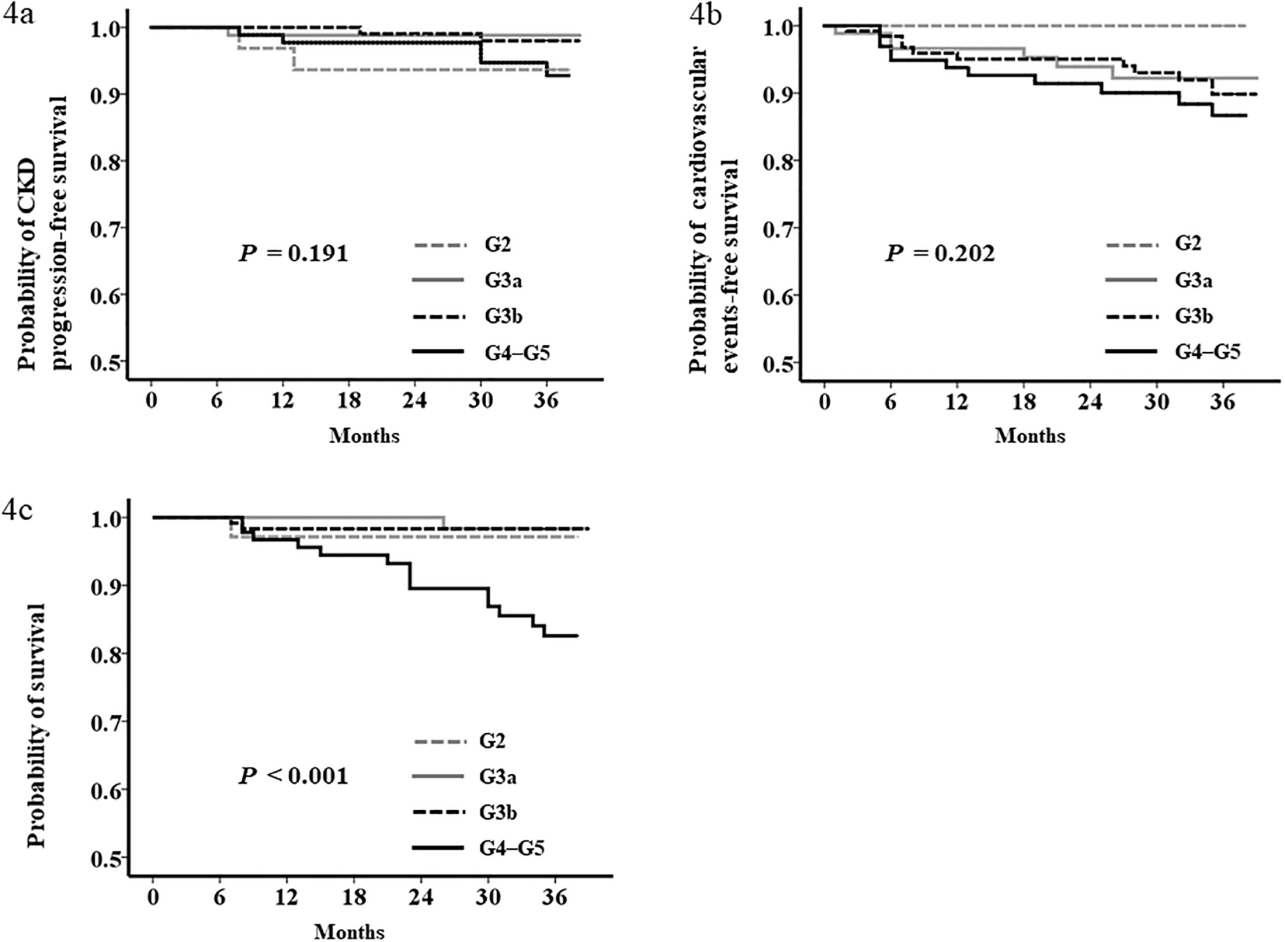
Kaplan–Meier curves for event-free survival in patients with normal-range proteinuria according to chronic kidney disease (CKD) stage (n = 352). Kaplan–Meier analysis demonstrated that chronic kidney disease (CKD) progression (Fig 4a) and development of cardiovascular events (Fig 4b) were not significantly different among CKD stages. All-cause death was significantly higher in stage G4–G5 CKD (Fig 4c).

Analysis for study endpoints revealed that 10 patients exhibited CKD progression (>50% eGFR loss, n = 9; initiation of dialysis, n = 1), 28 patients experienced cardiovascular events, and 18 patients died during the median follow-up of 36 months in this study (Table 4). The incidence rates of the three endpoints in all patients were 1.14, 3.34, and 2.06 per 100 person-years, respectively. In advanced CKD stage G4–G5, only 5.1% of patients had CKD progression. Kaplan–Meyer analysis demonstrated that CKD progression (*P* = 0.191) and cardiovascular events (*P* = 0.202) were not significantly different among CKD stages (Fig 4a and 4b). All-cause death was significantly higher in patients with G4 or G5 CKD (*P* < 0.001; Fig 4c). Table 5 shows Cox proportional hazard analysis for disease endpoints. Univariate analysis to determine the risk of disease endpoints showed that the G2, G3b, G4–G5 CKD patients were not at risk for CKD progression and development of cardiovascular events compared with G3a CKD patients, whereas G4–G5 patients were at a risk for all-cause death compared with G3a (HR 12.86; 95%CI 1.69–97.84). However, multivariate analysis showed that the risks of three endpoints were not significantly different among CKD stages in model 1 and model 2.

**Table 4:** Numbers and rates of incidence in patients with normal-range proteinuria (n = 352).

	CKD progression			Cardiovascular events			All-cause death		
	incidence		Rate, per 100 person-years	incidence		Rate, per 100 person-years	incidence		Rate, per 100 person-years
All (n = 352)	10	2.8%	1.14	28	8.0%	3.34	18	5.1%	2.06
G2 (n = 36)	2	5.6%	2.29	0	0%	0	1	2.8%	1.15
G3a (n = 89)	1	1.1%	0.45	6	6.7%	2.85	1	1.1%	0.45
G3b (n = 129)	2	1.6%	0.61	11	8.5%	3.51	2	1.6%	0.61
G4–G5 (n = 98)	5	5.1%	2.09	11	11.2%	4.84	14	14.3%	5.86

Abbreviations: CKD, chronic kidney disease.

**Table 5:** Cox proportional hazard model for risk of disease endpoints according to CKD stages (n = 352).

CKD progression	Unadjusted	Model 1	Model 2
G2	5.18 (0.47–57.13)	4.67 (0.41–53.64)	16.38 (0.62–435.49)
G3a	ref	ref	ref
G3b	1.33 (0.12–14.73)	1.49 (0.13–17.24)	3.81 (0.19–75.40)
G4–G5	4.63 (0.54–39.63)	5.94 (0.63–56.01)	8.21 (0.61–110.28)
Cardiovascular events	Unadjusted	Model 1	Model 2
G2	0	0	0
G3a	ref	ref	ref
G3b	1.22 (0.45–3.29)	0.67 (0.24–1.91)	0.67 (0.23–1.95)
G4–G5	1.69 (0.63–4.58)	0.70 (0.23–2.14)	0.88 (0.26–2.94)
All-cause death	Unadjusted	Model 1	Model 2
G2	2.55 (0.16–40.73)	4.81 (0.29–78.90)	5.93 (0.33–105.69)
G3a	ref	ref	ref
G3b	1.35 (0.12–14.91)	0.92 (0.08–10.51)	0.94 (0.08–11.36)
G4–G5	12.86 (1.69–97.84)	7.42 (0.87–63.50)	4.47(0.47–42.21)

Data are presented as hazard ratios (95% confidence intervals).

Model 1: Adjusted by age, gender, hypertension, diabetes, and history of CVD. Model 2, Adjusted by Model 1, hemoglobin, serum albumin, and the use of RAAS inhibitor. Abbreviations: CKD, chronic kidney disease.

## Discussion

In this observational cohort study, kidney function in patients with normal-range proteinuria was at a significantly lower risk for progression than that in patients with abnormal-range proteinuria. In patients with normal-range proteinuria, kidney function did not decrease over the course of 36 months, even if they had advanced-stage CKD. The risk of cardiovascular events did not differ significantly among CKD stages in the patients with normal-range proteinuria. Proteinuria has been proposed as a surrogate endpoint in clinical trials on CKD progression [11]. Evaluation of proteinuria is simple and inexpensive. Mass screening of 106,177 participants from the Japanese general population in Japan identified proteinuria as the most powerful predictor of ESKD risk over ten years [12]. Furthermore, among patients with diabetic nephropathy, baseline albuminuria was a strong independent predictor of progression to ESKD in the Reduction in End Points in Noninsulin-Dependent Diabetes Mellitus with the Angiotensin II Antagonist Losartan (RENAAL) study and the Irbesartan in Diabetic Nephropathy Trial (IDNT) [13, 14]. Thus, proteinuria is a marker of renal risk both in general population and CKD patients. However, few studies investigated disease endpoints in CKD patients with normal-range proteinuria who were stratified according to the disease stage. The percentage of such patients with eGFR <60 ml/min/1.73 m^2^ and negative or trace proteinuria by dipstick urine test was 32.5% in the current study (Table 1). In the US general population with eGFR < 60 ml/min/1.73m^2^, normo-albuminuria was observed in 66% cases [15]. In the German Chronic Kidney Disease cohort, which included 5,217 patients with a mean eGFR of 47.1 ml/min/1.73m^2^, 41.8% patients had eGFR < 60 ml/min/1.73m^2^ and urinary albumin/creatinine ratio (ACR) < 30 mg/g [16]. These reports suggested that there was a high prevalence of CKD patients with normal-range proteinuria, and these patients might be overlooked in health checkups. The findings of the current study suggest that the prognosis of kidney function and risks of cardiovascular events and mortality in these patients were unexpectedly not so high. Incidence rates of three endpoints (CKD progression, cardiovascular events, and all-cause death) in patients with normal-range proteinuria were 1.14, 3.34 and 2.06 per 100 person-years, respectively (Table 4). Similarly, it was reported that the incidence rates of ESKD and all-cause death in low protein (< 0.5 g/24 h) CKD G3–5 patients were 2.7 and 3.7 per 100 person-years, respectively [17].

Previous studies have reported that among whole CKD patients under specialized nephrology care, the dominant clinical outcome was ESKD rather than death [18, 19]. However, the incidence rate of all-cause death was higher than CKD progression in our study, showing an inverse result in outcome. Moreover, among the 10 patients who reached the composite outcome of CKD progression with >50% eGFR loss or initiation of dialysis, only one patient developed ESKD. This result possibly reflects the low risk of CKD progression of normo-proteinuric CKD patients.

One of the widely accepted mechanisms underlying proteinuria-mediated acceleration of progressive kidney injury includes tubulointerstitial damage precipitated by the direct toxicity of filtered urinary proteins. Recent studies support the possibility that the excessive protein accumulation in podocytes is one factor underlying progressive injury of glomerular cells through the release of transforming growth factor β, which ultimately allows myofibroblastic differentiation of mesangial cells [20]. The underlying pathophysiologic mechanism linking albuminuria and cardiovascular disease has been proposed as a renal manifestation of peripheral vascular dysfunction and specifically endothelial dysfunction, which may accelerate the atherothrombotic process and subsequently increase cardiovascular disease risk [21].

One of the potential factors contributing to the better outcomes in our study is the specialized care that the patients were receiving from nephrologists. Subjects enrolled in the current study were newly visiting patients or those who were referred from other institutions, many of whom received nutritional guidance (i.e., low salt and low protein diet) for the first time. As specialized nephrology care is associated with better prognosis in CKD patients [22–24], appropriate diet therapy is critical for protecting patients from disease progression.

In our daily clinical practice experience, often, CKD does not progress in patients without proteinuria. However, these experiences were not reported in newly visiting CKD patients. The current study demonstrated that CKD might not progress in patients with normal-range proteinuria even if they had advanced-stage CKD. This new finding is the major strength of the current study.

One noted feature of this study was the low prevalence of diabetic nephropathy (7.7%) as a CKD etiology in patients with normal-range proteinuria (Table 2). Among all the patients enrolled in the original CKD-ROUTE study, the prevalence of diabetic nephropathy was 25.5% [7], which is quite similar to that reported in other CKD cohort studies in Japan [25]. This finding indicates that diabetic nephropathy patients were not incidentally excluded at enrollment. In typical diabetic nephropathy, patients exhibit glomerular hyperfiltration with moderate albuminuria during the microalbuminuria phase in very early disease, followed by severely increased albuminuria during the macroalbuminuria phase as the disease progresses. The onset of albuminuria often precedes the progressive decline in GFR, which ultimately leads to end-stage kidney disease. Therefore, it can be speculated that CKD patients with concomitant renal dysfunction and normo-proteinuria (stages G2A1 to G5A1) are not as likely to have diabetic nephropathy as the disease etiology. In accordance with our observation, another cohort study of CKD with low-grade proteinuria has also reported a high prevalence of nephrosclerosis (39.8%) and a relatively low prevalence of diabetic nephropathy (11.3%) as the etiology of CKD [17].

The current study has several limitations. First, the etiology of CKD was determined by the attending doctor’s diagnosis. Many patients did not undergo renal biopsy. Second, the effect of diet therapy was not evaluated by a specialized nephrologist. Third, detailed information on lifestyle, such as smoking and drinking, was not collected or evaluated.

In summary, the kidney function of CKD patients with normal-range proteinuria did not decrease in the advanced stages of CKD. Additionally, the risks for cardiovascular events and mortality did not significantly differ among CKD stages in patients with normal-range proteinuria. These findings suggest that newly visiting CKD patients with normal-range proteinuria tend to be overlooked during health checkups and may not exhibit CKD progression even in advanced CKD stages under specialized nephrology care.

## Supporting information

S1 Table Classification of chronic kidney disease severity according to the Japanese guidelines. (DOCX)
