# Supplementary material for: Prognosis of chronic kidney disease with normal-range proteinuria: The CKD-ROUTE study
Source: PLoS One. 2018 Jan 17;13(1):e0190493. doi: 10.1371/journal.pone.0190493 (PMC5771558; doi:10.1371/journal.pone.0190493)
Supplement: S1 Table — (DOCX) [file pone.0190493.s001.docx]

S1 Table. Classification of chronic kidney disease severity according to the Japanese guidelines

|  |  |  | Proteinuria categories (urinary protein/creatinine ratio) | | | Total |
| --- | --- | --- | --- | --- | --- | --- |
|  |  |  | A1 | A2 | A3 |  |
|  |  |  | Normal | Mild proteinuria | Severe proteinuria |  |
|  |  |  | <0.15 | 0.15–0.5 | ≥0.5 |  |
|  |  |  | n (%) | n (%) | n (%) |  |
| GFR categories (mL/min/1.73 m^2^) | G2 | 60–89 | 38 (3.6) | 16 (1.5) | 36 (3.4) | 90 (8.6) |
|  | G3a | 45–59 | 87 (8.3) | 38 (3.6) | 53 (5.0) | 178 (17.0) |
|  | G3b | 30–44 | 92 (8.8) | 57 (5.4) | 106 (10.1) | 255 (24.3) |
|  | G4 | 15–29 | 64 (6.1) | 48 (4.6) | 224 (21.3) | 336 (32.0) |
|  | G5 | <15 | 6 (0.6) | 13 (1.2) | 172 (16.4) | 191 (18.2) |
| Total | | | 287 (27.3) | 172 (16.4) | 591 (56.3) | 1050 (100) |

Data are presented as numbers and percentages.

Abbreviations: GFR, glomerular filtration rate.
